# Lack of a-disintegrin-and-metalloproteinase ADAM10 leads to intracellular accumulation and loss of shedding of the cellular prion protein *in vivo*

**DOI:** 10.1186/1750-1326-6-36

**Published:** 2011-05-27

**Authors:** Hermann C Altmeppen, Johannes Prox, Berta Puig, Mark A Kluth, Christian Bernreuther, Dana Thurm, Ellen Jorissen, Bettina Petrowitz, Udo Bartsch, Bart De Strooper, Paul Saftig, Markus Glatzel

**Affiliations:** 1 Institute of Neuropathology, University Medical Center Hamburg-Eppendorf, D-20246 Hamburg, Germany; 2 Biochemical Institute, Christian-Albrechts University, D-24098 Kiel, Germany; 3 Department of Tumor Virology, Heinrich-Pette-Institute for Experimental Virology and Immunology, D-20251 Hamburg, Germany; 4 Center for Human Genetics, Katholieke Universiteit Leuven, Belguim; 5 Department for Developmental and Molecular Genetics, Vlaams Instituut voor Biotechnologie (VIB), 3000 Leuven, Belgium; 6 Department of Ophthalmology, University Medical Center Hamburg-Eppendorf, D-20246 Hamburg, Germany

## Abstract

**Background:**

The cellular prion protein (PrP^C^) fulfils several yet not completely understood physiological functions. Apart from these functions, it has the ability to misfold into a pathogenic scrapie form (PrP^Sc^) leading to fatal transmissible spongiform encephalopathies. Proteolytic processing of PrP^C ^generates N- and C-terminal fragments which play crucial roles both in the pathophysiology of prion diseases and in transducing physiological functions of PrP^C^. A-disintegrin-and-metalloproteinase 10 (ADAM10) has been proposed by cell culture experiments to be responsible for both shedding of PrP^C ^and its α-cleavage. Here, we analyzed the role of ADAM10 in the proteolytic processing of PrP^C ^*in vivo*.

**Results:**

Using neuron-specific *Adam10 *knockout mice, we show that ADAM10 is the sheddase of PrP^C ^and that its absence *in vivo *leads to increased amounts and accumulation of PrP^C ^in the early secretory pathway by affecting its posttranslational processing. Elevated PrP^C ^levels do not induce apoptotic signalling via p53. Furthermore, we show that ADAM10 is not responsible for the α-cleavage of PrP^C^.

**Conclusion:**

Our study elucidates the proteolytic processing of PrP^C ^and proves a role of ADAM10 in shedding of PrP^C ^*in vivo*. We suggest that ADAM10 is a mediator of PrP^C ^homeostasis at the plasma membrane and, thus, might be a regulator of the multiple functions discussed for PrP^C^. Furthermore, identification of ADAM10 as the sheddase of PrP^C ^opens the avenue to devising novel approaches for therapeutic interventions against prion diseases.

## Background

**Figure 1:**
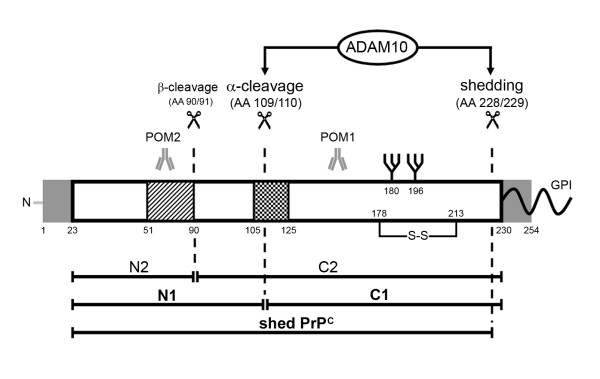
**Proteolytic cleavage of PrP**^**C**^. After cleavage of the N-terminal and GPI anchor signal peptides (grey), mature membrane-bound PrP^C ^can undergo at least three different proteolytic cleavage events (scissors) in addition to phospholipase C cutting within its GPI anchor. While β-cleavage is mainly observed under pathological conditions, a role of ADAM10 is discussed in the context of both, constitutive α-cleavage and shedding of PrP^C^. The resulting fragments are shown underneath. Striped box: octameric repeat region; checked box: hydrophobic core domain. Epitopes of PrP^C ^specific monoclonal antibodies POM1 and POM2 used in this study are indicated.

The cellular prion protein (PrP^C^), a glycosylphosphatidylinositol (GPI)-anchored membrane protein, plays a dual role in the biology of the brain. It is the substrate for the generation of its pathological isoform (PrP^Sc^), which is the principal component of prion infectivity and causally involved in the pathophysiology of prion diseases. On the other hand, it accomplishes a multitude of physiological functions ranging from neurogenesis to myelin maintenance [1,2]. Proteolytic processing of PrP^C ^generating N- and C-terminal cleavage products as well as shed full length PrP^C^, is critically involved in the above mentioned physiological and pathophysiological aspects [2-4]. For instance, a membrane-bound, C-terminal fragment of PrP^C ^is essential for myelin maintenance, and shed GPI-anchorless PrP^Sc ^modulates prion neuroinvasion and prion disease phenotypes [2,5]. While β-cleavage is mainly observed under pathological conditions, key proteolytic processing of PrP^C ^under physiological conditions includes α-cleavage resulting in a membrane-bound, C-terminal (C1) and a soluble, N-terminal (N1) fragment, and ectodomain shedding of PrP^C^, releasing a GPI-anchorless form into the extracellular space [6-10] (Figure 1). A number of target proteases have been implicated in α-cleavage and ectodomain shedding of PrP^C ^under *in vitro *conditions with a-disintegrin-and-metalloproteinase (ADAM) 10 and ADAM17 as prime candidates for both cleavage events [8,10].

ADAM10 mediates the shedding of several surface proteins in close proximity to the cellular membrane. Substrates of ADAM10 in the brain include ephrins [11-13], neuronal adhesion molecule [14], N-cadherin [15], and the Notch receptor [16-19]. Initially demonstrated in cell culture experiments, ADAM10 has recently been identified as the α-secretase of the amyloid precursor protein (APP) *in vivo *[20-23]. Involvement of ADAM10 in α-cleavage and shedding of PrP^C ^has been studied in cell culture leading to some controversy [8,10]. Furthermore, a complete *Adam10 *knockout in mice resulted in early embryonic lethality making it impossible to study its influence on neuronal PrP^C ^processing *in vivo *[18]. This problem was overcome by the recent generation of a conditional, neuron-specific *Adam10 *knockout mouse (hence ADAM10 cKO or A10 cKO) [21]. Here, we used these ADAM10 cKO mice to study the effects of ADAM10 on proteolytic processing of PrP^C^. We show that ADAM10 is responsible for ectodomain shedding of PrP^C ^and that its absence results in PrP^C ^accumulation in the early secretory pathway. Additionally, we demonstrate that ADAM10 is not responsible for α-cleavage of PrP^C^.

## Results

### Lack of ADAM10 leads to posttranslational increase of PrP^C^

**Figure 2:**
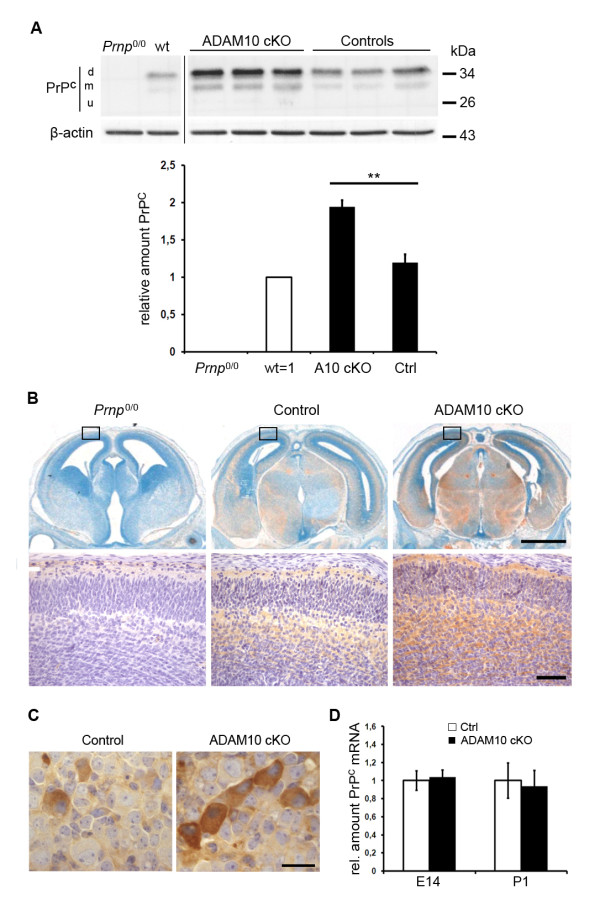
**Increased amounts of PrP**^**C **^**but unaltered mRNA levels in ADAM10 cKO mice**. (A) Western blot analysis for PrP^C ^in primary neurons of *Prnp*^0/0^, wt, ADAM10 cKO and *Nestin-Cre-*negative littermate controls (all from E14 embryos). Quantification of PrP^C ^amounts shows a statistically significant increase of PrP^C ^in ADAM10 cKO neurons compared to littermate controls (*p *= 0,0013). All samples were normalized to wildtype which was set to one (n = 3 for wt and *Prnp*^0/0^; n = 5 for A10 cKO and Ctrl) (d, m, u = di-, mono-, unglycosylated PrP^C^; representative blot is shown). Immunohistochemical staining of PrP^C ^in brains (B) and dorsal root ganglia (C) of E14 ADAM10 cKO mice and littermate controls shows enhanced immunosignal throughout the entire central nervous system with some degree of variation between individual neurons in ADAM10 cKO mice (C, scale bar: 20 μm). Brain of an age-matched *Prnp*^0/0 ^mice was taken as negative control in B. (B, upper row: whole brain section, scale bar: 500 μm; bottom row: magnification of inserts, scale bar: 50 μm). (D) Quantitative RT-PCR for PrP^C ^mRNA in brain homogenates of ADAM10 cKO and wildtype littermate controls shows no significant difference in expression levels between genotypes at E14 (n = 4) and P1 (n = 3).

Indirect evidence suggested that ADAM10 modulates PrP^C ^levels [24]. A recently published, novel conditional knockout model lacking ADAM10 specifically in neurons allowed us to assess this notion *in vivo *[21]. Here, we used primary neurons and brain sections of ADAM10 cKO mice at embryonic day 14 (E14) to investigate the expression and localisation of PrP^C^. Lack of ADAM10 resulted in increased levels of PrP^C ^as shown by Western blot analysis (Figure 2A). After quantification, PrP^C ^amounts were increased by a factor of 1.94 (standard error of mean, SEM: 0,096) when compared to PrP^C ^levels in neuronal lysates derived from age-matched wildtype embryos (set as one), with *Nestin-Cre-*negative littermates (Controls) showing values of 1.19 (SEM: 0,117). Thus, PrP^C ^levels were increased by 63% in ADAM10 cKO compared to littermate controls and differences were significant (*p *= 0,0013). Assessment of the contribution of di-, mono- and unglycosylated PrP^C ^to the total signal of PrP^C ^in Western blot analysis of ADAM10 cKO and control neurons revealed comparable glycoprofiles showing that ADAM10-related PrP^C ^increase did not alter the extent of glycosylation of PrP^C ^(data not shown). In accordance with published data, morphological analysis of ADAM10 cKO mice and controls at E14 did not show alterations in the gross cortical organisation [21]. An enhanced immunosignal for PrP^C ^was observed in all cortical and subcortical regions (Figure 2B) as well as in the spinal cord including sensory ganglia when compared to littermate controls with a staining pattern showing increased signal intensity in neurons of the central (Figure 2B) and peripheral nervous system (Figure 2C). Since we were interested in defining whether the increase in PrP^C ^is due to transcriptional upregulation or due to posttranslational mechanisms, we quantified neuronal PrP^C ^mRNA levels by quantitative RT-PCR. No significant differences were detectable between wildtype littermate controls (set as one) and ADAM10 cKO neurons when assessed at E14 (A10 cKO: relative value = 1,037; SEM = 0,078; SEM Ctrl = 0,160; *p *= 0,79) or postnatal day 1 (A10 cKO: rel. value = 0,935; SEM = 0,175; SEM Ctrl = 0,195; *p *= 0,82) (Figure 2D). Thus, increased PrP^C ^levels in brains and primary neurons of ADAM10 cKO mice are not caused by transcriptional upregulation but are rather due to posttranslational mechanisms.

### Lack of ADAM10 leads to accumulation of PrP^C ^in the early secretory pathway

**Figure 3:**
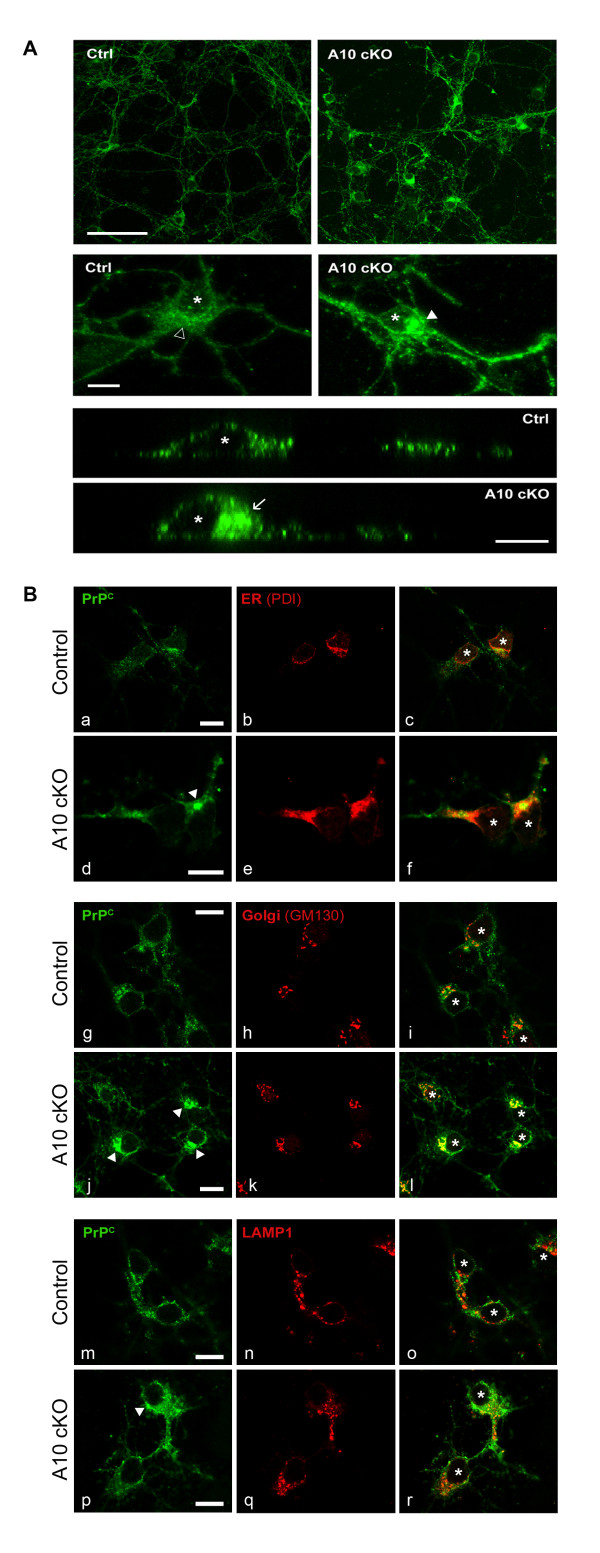
**PrP**^**C **^**accumulates in intracellular compartments in ADAM10 cKO neurons**. (A) Confocal (upper two rows; scale bar overview: 100 μm, scale bar single neuron: 10 μm) and spinning disc (lower two rows; scale bar YZ-sections: 10 μm) immunofluorescence microscopy for PrP^C ^in permeabilized wildtype neurons (Ctrl) shows PrP^C ^in evenly distributed vesicular structures (open arrowhead). In ADAM10 cKO neurons, dense PrP^C ^accumulations are detectable adjacent to the nucleus (filled arrowhead) and in neuronal processes. YZ-sections through individual neurons confirm these findings (arrow for PrP^C ^accumulation in ADAM10 cKO). Asterisks mark nuclei. (B) Confocal microscopic analysis of co-stainings of PrP^C ^(left column) with intracellular organelle marker proteins (middle column) in wildtype littermate (Control) and ADAM10 cKO neurons. Overlays are shown in the right column. Accumulations of PrP^C ^are only found in ADAM10 cKO neurons (filled arrowheads in d, j, and p) and colocalize with protein disulfide isomerase (PDI) used as marker for ER (e, f) and Golgi marker GM130 (k, l). Partial colocalization of PrP^C ^with the lysosomal marker LAMP1 is detectable in vesicular structures (q, r). Scale bars represent 10 μm. Asterisks in overlays mark nuclei.

PrP^C ^is present both in intracellular compartments and on the plasma membrane. Thus, we were interested in examining the subcellular distribution of PrP^C ^and in locating its increase in ADAM10 cKO compared to wildtype littermate neurons. To this aim, we performed confocal and spinning disc immunofluorescence microscopy using a PrP^C^-specific antibody. Staining intensities of PrP^C ^at the plasma membrane appeared unaltered in ADAM10 cKO neurons when compared to controls (data not shown). However, in permeabilized control neurons, PrP^C ^was localized in vesicular structures that were evenly distributed within the soma, whereas in ADAM10 cKO neurons, dense PrP^C ^accumulations were found in intracellular compartments adjacent to the nucleus and along neuronal processes (Figure 3A). To determine the localization of these dense PrP^C ^accumulations in more detail we performed co-stainings of PrP^C ^with organelle markers in ADAM10 cKO neurons and controls. Control neurons showed a partial, vesicular colocalization with all three markers, reflecting the biosynthesis, trafficking and degradation of PrP^C^. Again, dense aggregates of PrP^C ^were only found in ADAM10 cKO neurons. These accumulations of PrP^C ^colocalized with ER marker PDI and with Golgi marker GM130 (Figure 3B). No significant colocalization of dense PrP^C ^accumulations, but a partial, vesicular colocalization of PrP^C ^with lysosomal marker LAMP1 was found, reflecting the physiological turnover of PrP^C^. These findings identify retention of PrP^C ^within the early secretory pathway rather than an interrupted degradation as the cause for increased PrP^C ^levels seen in the absence of ADAM10.

### Expression and activation of p53 is not altered by increased levels of PrP^C ^in ADAM10 cKO

**Figure 4:**
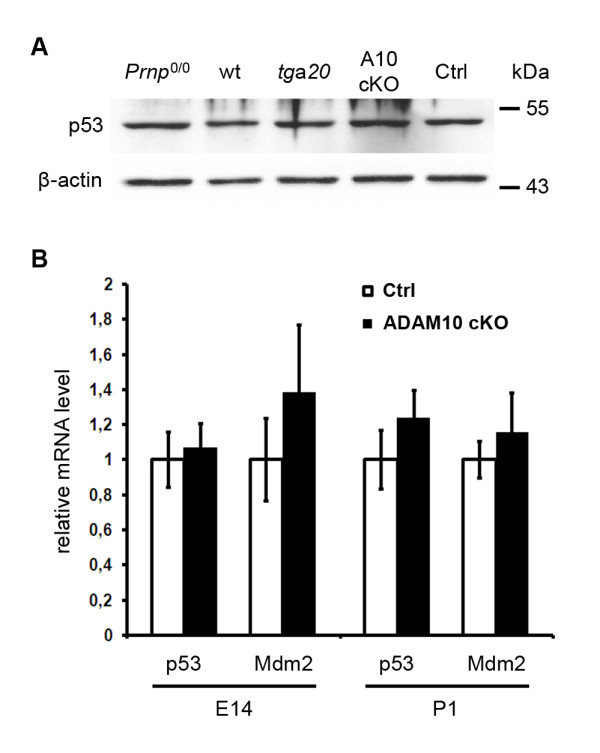
**No induction of p53 or p53-regulated pathways in ADAM10 cKO**. (A) Western blot analysis in lysates of primary neurons from *Prnp*^0/0^, wt, *tg*a*20*, ADAM10 cKO and littermate controls (all from E14 embryos) shows comparable protein amounts of p53. (B) Using quantitative RT-PCR, relative mRNA levels of p53 and its downstream signalling target Mdm2 were determined in brain homogenates of ADAM10 cKO mice and wildtype littermates controls at E14 (n = 4) and P1 (n = 3). No significant differences in expression levels of p53 and Mdm2 were observed between genotypes at both ages (E14: *p *= 0,75 for p53 and *p *= 0,44 for Mdm2; P1: *p *= 0,37 for p53 and *p *= 0,56 for Mdm2).

Increased expression of PrP^C ^has been shown to induce p53-related pathways [25,26]. Therefore, we decided to investigate p53-related signalling in our model of ADAM10-related PrP^C ^increase. Interestingly, we did not observe any evidence for induction of p53 at the protein level by Western blot analysis of neuronal lysates (Figure 4A). These data were confirmed by quantitative RT-PCR for p53 mRNA where there was no significant difference between ADAM10 cKO and littermate controls (set as one) when assessed at E14 (A10 cKO: rel. value = 1,07; SEM = 0,13; SEM Ctrl = 0,16; *p *= 0,75) and P1 (A10 cKO: rel. value = 1,24; SEM = 0,16; SEM Ctrl = 0,17; *p *= 0,37) (Figure 4B). In order to study a possible regulatory influence of increased PrP^C ^on p53-dependent signalling, we also analyzed mRNA levels of Mdm2 (Figure 4B) and p21 (encoded by the *Cdkn1a *gene; data not shown), two essential co-regulated targets in p53-related pathways [27]. No significant differences between neurons of ADAM10 cKO and littermate controls were detected (Mdm2 at E14: A10 cKO: rel. value = 1,38; SEM = 0,39; SEM Ctrl = 0,24; *p *= 0,44/Mdm2 at P1: A10 cKO: rel. value = 1,16; SEM = 0,22; SEM Ctrl = 0,11; *p *= 0,56). Thus, an ADAM10-related increase in PrP^C ^unlikely leads to the activation of p53-related pathways.

### α-cleavage of PrP^C ^in ADAM10 cKO mice

**Figure 5:**
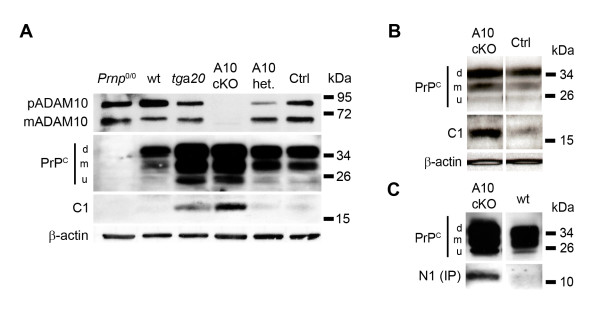
**ADAM10 is not responsible for α-cleavage of PrP**^**C**^. (A) Western blot analysis for premature (pADAM10) and mature ADAM10 (mADAM10; upper row), PrP^C ^(second row), PrP^C ^C1 fragment after PNGase F treatment (third row), and actin (bottom row) in neuronal cultures from *Prnp*^0/0^, wt, *tg*a*20*, ADAM10 cKO and littermate controls at E14. Levels of ADAM10 are dramatically reduced in ADAM10 cKO when compared to cultures from littermate controls or from mice overexpressing or lacking PrP^C^. In accordance with figure 2, PrP^C ^levels are increased in ADAM10 cKO and there is no influence of ADAM10 status on the presence of the C1 fragment, which is most abundant in neuronal lysates with elevated expression of PrP^C ^(i.e. ADAM10 cKO and *tg*a*20*). (B) Western blot analysis for PrP^C ^in brain of ADAM10 cKO and littermate controls at E14 confirms increased PrP^C ^and the presence of the C1 fragment in ADAM10 cKO mice in an *in vivo *setting. (C) Western blot analysis for PrP^C ^in lysates of ADAM10 cKO and wildtype neurons (both from E14 mice embryos) confirms increased PrP^C ^amounts in the absence of ADAM10. Soluble N1 fragment was immunoprecipitated (IP) from culture supernatants of these neurons and is found to be increased in supernatants derived from ADAM10 cKO neurons.

It was previously suggested that ADAM10 is responsible for the α-cleavage of PrP^C^, giving rise to the membrane-bound C1 and the soluble N1 fragment (Figure 1) [8,9]. Our ADAM10 cKO mice allowed us to investigate this hypothesis in primary neurons and total brain homogenates. First, we analyzed via immunoblotting the *Adam10 *deletion efficiency in neuronal lysates from ADAM10 cKO, ADAM10 littermate controls, wildtype, *tg*a*20*, and *Prnp*^0/0 ^mice (Figure 5A). ADAM10 cKO neurons showed a nearly complete loss of ADAM10 with heterozygous littermates (A10 het.) showing a reduction in the expression of both premature (pADAM10) and mature ADAM10 (mADAM10) when compared to *Nestin-Cre*-negative littermate controls (Ctrl), indicating a dose-dependency in gene expression (Figure 5A; lanes 4-6). The relative increase of pADAM10 in wildtype neurons was most likely due to differences in the efficiency of neuronal lysis and was not seen in neuronal lysates from different wildtype mice (Additional file 1A). Analysis further revealed that lack (*Prnp*^0/0^) or transgenic overexpression of PrP^C ^(*tg*a*20*) did not modulate the levels of pADAM10 or mADAM10. Again, Western blot analysis for PrP^C ^confirmed the increase of PrP^C ^in ADAM10 cKO neurons described above. Upon deglycosylation with PNGase F, C1 became detectable as a band of approx. 16 kDa. We could detect C1 irrespectively of the ADAM10 status of neurons (Figure 5A; complete Western blot of PNGase F digestion is shown in Additional file 1B). In fact, C1 signal strength was stronger in *tg*a*20 *and ADAM10 cKO neurons arguing that the amount of PrP^C ^rather than the presence of ADAM10 correlates with the appearance of C1. Increased levels of PrP^C ^and C1 were also found in brain homogenates of ADAM10 cKO mice at E14 when compared to wildtype littermate controls (Figure 5B). Furthermore, the corresponding, soluble N1 fragment of approx. 11 kDa was immunoprecipitated from supernatants of primary neurons and was present in all samples except for *Prnp*^0/0 ^neurons (Figure 5C and Additional file 1C). As with C1, the N1 fragment appeared to be increased in supernatants of ADAM10 cKO neurons when compared to wildtype controls. In summary, α-cleavage occurs in the absence of ADAM10, challenging the role of ADAM10 in this processing step of PrP^C ^*in vivo*.

### ADAM10 cKO mice do not shed PrP^C^

**Figure 6:**
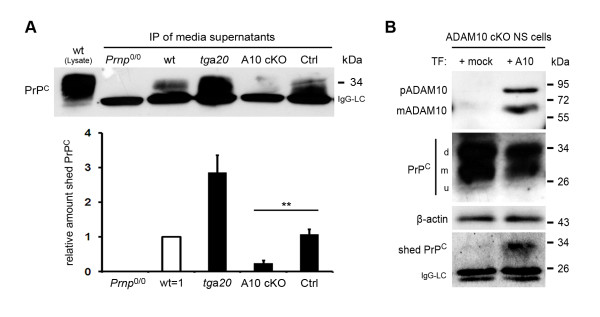
**ADAM10 is responsible for the shedding of PrP**^**C**^. (A) Shed PrP^C ^was immunoprecipitated (IP) from culture supernatants of primary neurons derived from *Prnp*^0/0^, wt, *tg*a*20*, ADAM10 cKO and littermate control mice (all from E14 embryos) and visualized by Western blot analysis for PrP^C^. Shed PrP^C^, which shows a 2-3 kDa shift when compared to PrP^C ^in lysates, is only detectable in wt, *tg*a*20*, and littermate control neurons. Supernatants from ADAM10 cKO neuron cultures contain virtually no shed PrP^C^. IgG light chain (IgG-LC) of capture antibody is detectable at 25 kDa (representative blot is shown). Quantification confirms a significant reduction (*p *= 0,0026) of the amounts of shed PrP^C ^shedding in ADAM10 cKO compared to littermate control cultures (*Prnp*^0/0 ^values were defined as background and subtracted from the values of all other genotypes; wildtype values were set to one; n = 3 for *tg*a*20*; n = 5 for A10 cKO and Ctrl). (B) Neural stem (NS) cells derived from an ADAM10 cKO mouse embryo at E14 were transfected (TF) without (+mock) or with *Adam10*-ORF (+A10). Following neuronal differentiation, PrP^C ^was immunoprecipitated from culture supernatants (bottom row; IgG-LC = IgG light chain of capture antibody) and detected in corresponding cell lysates (second row; d, m, u = di-, mono-, unglycosylated PrP^C^) by Western blotting. Shed PrP^C ^reappears in *Adam10*- but not in mock-transfected cultures (bottom row). Success of transfection was verified by Western blot detection of pADAM10 and mADAM10 in cell lysates (top row). Actin is shown as control (third row).

ADAM10 plays important roles in regulated intramembrane proteolysis and *in vitro *studies suggest that ADAM10 is involved in shedding of PrP^C ^at the plasma membrane [10]. Here, we investigated this issue using ADAM10 cKO primary neurons and performing immunoprecipitations of shed PrP^C ^from conditioned media. Following SDS-PAGE and immunoblotting, we observed an almost complete lack of shed PrP^C ^in culture supernatants from ADAM10 cKO neurons (Figure 6A; *tg*a*20*: rel. value = 2,85; SEM = 0,49; A10 cKO: rel. value = 0,24; SEM = 0,08; Ctrl: rel. value = 1,07; SEM = 0,15; *p *= 0,0026). An additional blot for shed PrP^C ^is shown in Additional file 1C). Immunoprecipitated shed PrP^C ^showed a slightly different running behaviour in gel electrophoresis when compared to PrP^C ^in neuronal lysates. This shift of 2 kDa reflects the truncation of shed PrP^C^, which lacks the GPI anchor and two C-terminal amino acids. To determine whether the lack of shedding is directly linked to the absence of ADAM10, we carried out rescue experiments by expressing ADAM10 in neural stem (NS) cells derived from ADAM10 cKO mice, followed by neuronal differentiation. Importantly, re-expression of ADAM10 led to reappearance of shed PrP^C ^in supernatants of neuronally differentiated NS cell cultures transfected with *Adam10*, but not in supernatants of mock-transfected control cultures (Figure 6B). Based on these results, we conclude that ADAM10 is both necessary and sufficient to release PrP^C ^from the neuronal surface.

## Discussion

PrP^C ^is in the focus of research because of its unique ability to act as an infectious agent in its misfolded form [28,29]. Furthermore, its physiological functions are manifold ranging from neurogenesis to binding of beta-amyloid [1,30]. Both aspects are influenced by proteolytic processing of PrP^C ^(Figure 1A; reviewed in [7]). Shedding at the plasma membrane, α-cleavage within the hydrophobic core, and β-cleavage subsequent to the octameric repeat region have been described for PrP^C ^[6,8,31-33]. However, all studies aimed at identifying the respective proteases have been performed using cell lines. For α-cleavage and shedding, the metalloproteinase ADAM10 has been suggested as the responsible protease [8-10]. Neuron-specific conditional ADAM10 knockout mice allowed us to address the role of ADAM10 for PrP^C ^processing *in vivo *[21].

Since a causal relationship between ADAM10 levels and PrP^C ^amounts has been postulated, we measured PrP^C ^levels in ADAM10 cKO mice [24]. We found a significant increase in PrP^C ^protein levels in ADAM10 cKO mice, while mRNA levels remained unaltered. Microscopic analysis showed an ADAM10-dependent intracellular accumulation of PrP^C ^in perinuclear compartments, but an unaltered expression pattern at the neuronal cell surface. This observation indicates that posttranslational mechanisms rather than transcriptional upregulation account for elevated PrP^C ^levels in the absence of ADAM10 and contrasts a study where overexpression of ADAM10 led to downregulation of PrP^C ^at the mRNA level [24]. Since we found PrP^C ^accumulations to be located in the ER and Golgi apparatus, we postulate that PrP^C ^is retained within these organelles in cells lacking ADAM10. In view of unaltered mRNA levels, constitutive biosynthesis in combination with the strong retention in the early secretory pathway seems to be the main reason for increased PrP^C ^levels in ADAM10 cKO mice.

PrP^C ^overexpression has been shown to downregulate beta-site APP-cleaving enzyme 1 (BACE1) in a glycosaminoglycan-dependent manner [34]. Interestingly, ADAM10 cKO mice show reduced APP-processing activity of BACE1 [21]. Thus, the data presented here provide a rationale for this phenomena linking ADAM10-related increase of PrP^C ^to reduction of BACE1 activity.

The interaction of PrP^C ^and p53 has been highlighted recently with overexpression of PrP^C ^leading to induction of p53-dependent pro-apoptotic pathways and p53 controlling expression of PrP^C ^via promoter transactivation [25,26,35,36]. In view of the fact that ADAM10 cKO mice die perinatally and show a degree of neurodegeneration that can only partially be explained by altered Notch signalling [21], PrP^C^-related induction of p53-dependent apoptosis seemed an attractive mechanism to explain this phenotype, in particular because of the increased amount of PrP^C ^found in these animals. However, we were unable to detect any dysregulation of p53-related pathways in our ADAM10 cKO neurons, which is in contrast to the study by Liang *et al. *who found a link between inducible upregulation of PrP^C ^and p53-dependent apoptosis [25,26,35,36]. Rather, our results are in line with a recent study showing cellular imbalance but unaltered p53 levels in response to PrP^C ^overexpression [37]. A recently published model states that p53 transcription is upregulated by the formation of the amyloid intracellular domain (AICD) during presenilin-dependent γ-secretase cleavage of APP with increased p53 subsequently activating PrP^C ^transcription [36]. Since production of the APP C-terminal fragment as a prerequisite for AICD formation is reduced in ADAM10 cKO mice [21], our findings of unaltered PrP^C ^and p53 mRNA levels fit into this concept.

The α-cleavage of PrP^C^, which takes place in the late secretory pathway [38], has been shown to be of utmost functional importance. Firstly, N-terminally truncated forms of PrP^C ^lead to neurodegeneration in transgenic mice [39-41]. Secondly, the N1 fragment can counteract experimentally induced p53-dependent caspase-3 activation *in vitro *and *in vivo*, indicating a neuroprotective function [42]. Thirdly, the corresponding C1 fragment was shown to act *in trans *on adjacent Schwann cells maintaining myelination [2], and it has been proposed to activate apoptotic pathways leading to neuronal death [3]. The α-cleavage occurs within the hydrophobic core, one of the most highly conserved domains of PrP^C^, which underlines the importance of this processing [43]. Thus, elucidating the nature of the responsible protease, recently termed α-PrPase, will help in understanding the physiological functions of PrP^C ^and the pathophysiology of prion disease [6,44,45]. Since ADAM10 was suggested to be the α-PrPase [8,9,46], we assessed this in our model. The fact that we found increased levels of C1 and N1 in primary neurons and total brain homogenates of ADAM10 cKO embryos clearly argues against involvement of ADAM10 in α-cleavage of PrP^C ^with increased amounts of PrP^C ^in ADAM10 cKO yielding elevated levels of C1 and N1. Although a varying PNGase F digestion efficiency could partially contribute to enhanced C1 presence in A10 cKO neurons compared to *tg*a*20 *in Figure 5A, we speculate that ADAM10 negatively regulates α-cleavage possibly by inhibiting the responsible protease. Our findings are in line with *in vitro *results of Taylor and colleagues, who were also unable to directly link ADAM10 expression or silencing to C1 prevalence [10]. Thus, the α-PrPase, which has recently been shown to tolerate severe modifications within the PrP^C ^sequence [45], still needs to be identified.

ADAM10 is the sheddase of a number of transmembrane proteins and was linked to constitutive shedding of PrP^C ^using cell lines overexpressing PrP^C ^[10,47]. Here, we investigated the role of ADAM10 in PrP^C ^processing in a physiological context *in vivo*. In agreement with *in vitro *data, we found an almost complete lack of PrP^C ^shedding in ADAM10 cKO mouse neurons. Furthermore, genetic introduction of *Adam10 *into ADAM10 cKO NS cells rescued shedding activity. Thus, we were able to identify ADAM10 as the functionally relevant sheddase of PrP^C^, which is further supported by the increase in PrP^C ^protein levels in ADAM10 cKO mice discussed above. Based on our results, we propose a model where shedding by ADAM10 is a mechanism to regulate the PrP^C ^amount at the cellular membrane. When shedding is impaired, the cell reacts with retention of PrP^C ^in the early secretory pathway rather than allowing it to accumulate at the plasma membrane. In view of the multiple functions discussed for PrP^C^, our findings indicate the importance of tightly controlled amounts of PrP^C ^at the neuronal surface.

## Conclusion

Our data show that ADAM10 does not perform α-cleavage but shedding of PrP^C^, with lack of ADAM10 activity leading to increased amounts of PrP^C ^due to its retention within early secretory compartments. These data further underscore the physiological relevance of proteolytic processing of PrP^C ^and shed light on the nature of the PrP^C^-sheddase. Yet, its impact on the course of prion disease is not known in detail. While a recent *in vitro *study showed no shedding-dependent modulation of prion conversion [10], incubation times after scrapie inoculation were prolonged in mice overexpressing *Adam10 *[24]. Further studies of proteolytic processing of PrP^C ^may provide novel approaches for therapeutic interventions against prion diseases.

## Methods

### Generation of murine primary neuronal cultures

Primary neurons were obtained from E14 embryos of *Adam10 *conditional knockout mice (*Adam10*^*Fl/Fl *^*Nestin-Cre*-positive; ADAM10 cKO or A10 cKO) [21,48]. *Adam10*^*Fl/+ *^*Nestin-Cre*-positive (A10 heterozygous) and *Adam10*^*Fl/+ *^*Nestin-Cre-*negative (A10 wildtype control or Ctrl) embryos served as littermate controls. Further controls included prion protein knockout embryos (*Prnp*^0/0^; [49]), wildtype embryos (wt; C57BL/6), and prion protein overexpressing embryos (*tg*a*20*; [50]). Briefly, embryonic brains were isolated, trypsinized, and cell suspensions were seeded on poly-L-lysine coated (Sigma) cell culture dishes (Sarstedt) in B-27 containing neurobasal medium (Invitrogen) as described previously [51]. Non-neuronal cells were eliminated by treatment with 5 μM cytosine arabinoside (Sigma) 24 h after plating. After four days, primary neurons were cultivated for additional 24 h in medium lacking B-27. Culture supernatants were collected for immunoprecipitation experiments and cells were lysed for Western blot analysis as described below. All animal procedures were performed in accordance with the institutional guidelines from the animal facility of the University Medical Center Hamburg-Eppendorf.

### Immunoprecipitation of PrP^C ^from conditioned media

Protease inhibitor cocktail (PI; Roche) was added to the supernatants. After centrifugation for 5 min at 3,000 × g and 4°C, volumes were normalized according to the total protein content of corresponding cell lysates (see below) and supernatants (approx. 4 ml) were concentrated by use of Amicon Ultra centrifugal filters 3 kDa MWCO (Millipore) at 5,000 × g and 4°C. 2 μg of mouse monoclonal antibody POM2 (A. Aguzzi, Zürich, Switzerland), recognizing repetitive epitopes within the N-terminus of PrP^C^, were added to 500 μl of concentrated supernatants, and antibody binding was allowed for 16 h at 4°C on a rotating wheel. Protein G sepharose beads "4 Fast Flow" (GE Healthcare) were washed 3× with RIPA buffer (50 mM Tris-HCl pH8, 150 mM NaCl, 1% NP40, 0,5% Na-Deoxycholat, 0,1% SDS) containing PI. 50 μl of a 1:1 suspension of beads in RIPA buffer was then added to the supernatant-antibody solution. After 1 h of incubation under rotation at 4°C, complexes were washed with RIPA buffer and boiled for 6 min with 2× loading buffer. Eluates were subsequently separated from the beads by centrifugation and then subjected to SDS-PAGE as described below.

### Western blot analysis of primary neuronal cultures and brain homogenates

Primary neurons were washed 2× with ice-cold PBS (PAA laboratories) and then lysed in RIPA buffer containing PI. Centrifugation for 5 min at 12,000 × g and 4°C was performed to get rid of DNA and cellular debris. Brain homogenates were prepared as described previously [52]. Protein concentrations were determined by Bradford assay (Biorad). For detection of the PrP^C ^C1 fragment, 30 μg of total protein was resuspended in digestion buffer (25 mM Tris-HCl pH7.5, 0,5% SDS, 1% β-mercaptoethanol) and boiled for 5 min. 1% Nonident P-40 (Fluka) and 5U of N-glycosidase F (PNGase F; Roche) were added and deglycosylation was allowed for 16 h at 37°C. Finally, samples were mixed with 4× loading buffer (250 mM Tris-HCl pH6.8, 8% SDS, 40% glycerol, 20% β-mercaptoethanol, 0,008% bromphenol blue), boiled for 6 min at 95°C, and electrophoretically separated using 12% Tris/glycine gels. Proteins were transferred to nitrocellulose membranes (Biorad) and detected using mouse monoclonal antibodies to PrP^C ^(POM1, 1:2,500 and POM2, 1:5,000; A. Aguzzi, Zürich, Switzerland) or to β-actin (1:5,000; Sigma), rabbit monoclonal antibody to p53 (1:1,000; Santa Cruz), and rabbit polyclonal antibody to ADAM10 (P. Saftig, Kiel, Germany), as well as the respective anti-mouse or anti-rabbit secondary antibodies (1:5,000; Promega). For immunoprecipitated N1 fragment, POM2 was used for pull-down and detection. Blots were developed with SuperSignal West Pico (Pierce). Quantifications were performed using Universal Hood II and Quantity One 4.6.2 software (Biorad).

### Generation, nucleofection, and differentiation of neural stem cell cultures

To derive adherently growing neural stem (NS) cells from ADAM10 cKO mice, we first established neurosphere cultures from the ganglionic eminence of E14 embryos using standard protocols (see e.g. [53]). Neurospheres from the third passage were enzymatically dissociated using Accutase (PAA Laboratories), cells were plated into gelatine-coated tissue culture flasks and further cultivated in NS-A medium (Euroclone) supplemented with 10 ng/ml fibroblast growth factor-2 (FGF-2), 10 ng/ml epidermal growth factor (EGF) (both from TEBU) and 1% modified N2 [54]. To express ADAM10 in ADAM10 cKO NS cells, mouse *Adam10 *cDNA was cloned into pcDNA3.1/Zeo(-) (Invitrogen), and the plasmid was linearized with Bgl II and used to transfect the cells with the Nucleofector^® ^technology (Lonza) as described [55]. Mock transfections were performed with linearized pcDNA3.1/Zeo(-) lacking the *Adam10 *cDNA. In brief, about 5 × 10^6 ^NS cells were resuspended in 100 μl Nucleofector^® ^solution containing 10 μg of linearized DNA, and cells were nucleofected using the Nucleofector^® ^program A033. Transfected cells were plated into tissue culture flasks coated with poly-L-ornithine and 1% Matrigel (Becton Dickinson), and expanded in NS-A containing 10 ng/ml FGF-2, 10 ng/ml EGF, 1% B27 (Invitrogen), 1% modified N2 and 200 μg/ml zeocin. To induce neuronal differentiation of ADAM10- and mock-nucleofected ADAM10 cKO NS cells, cells were first cultivated for four days in NS-A supplemented with 5 ng/ml FGF-2, 2% B27, and 1% modified N2, followed by another four days in a 1:1 mixture of NS-A and Neurobasal (Invitrogen) supplemented with 2% B27 and 0.25% modified N2. Supernatants from differentiated cultures were collected and subjected to immunoblot analysis.

### Sample preparation and immunohistochemical analysis

Embryos were dissected and fixed by immersion in 4% buffered formalin for 24 h, dehydrated in ascending ethanol concentrations, and embedded in low-melting-point paraffin following standard laboratory procedures. 4 μm sections were submitted to immunostaining following standard immunohistochemistry procedures using the Ventana Benchmark XT machine (Ventana). Briefly, deparaffinated sections were boiled for 30 min in CC1 buffer (Ventana) for antigen retrieval. Sections were then incubated with primary antibody POM1 (1:100) in 5% goat serum (Dianova), 45% Tris buffered saline pH7.6, 0,1% Triton X-100 in antibody diluent solution (Zytomed) for 1 h, followed by detection with Mouse Stain Kit (Nichirei Biosciences) for the detection of murine primary antibodies on murine tissue. Antibody detection was performed with an ultraview universal DAB detection kit (Ventana) followed by counterstaining according to the standard settings of the machine. As negative controls primary antibodies were omitted.

### Quantitative RT-PCR

RNA was extracted from entire brains of E14 (n = 4) and P1 (n = 3) *Adam10 *cKO and littermate control animals using the NucleoSpin RNAII kit (Macherey Nagel). RNA concentration was determined using the NanoDrop system (Thermo Scientific). First-strand cDNA was synthesized using 1 μg of total DNase-treated RNA in a 20 μl reverse transcriptase reaction mixture following the instructor manual (High Capacity cDNA Reverse Transcription Kit; Applied Biosystems). All Real-Time PCR reactions were performed in a 10 μl mixture containing 10 ng of cDNA preparation (1 μl), 2XSYBR^® ^Green PCR Master Mix (Applied Biosystems), and 0.2 μM of each primer. The following primer pairs were used: murine *Prnp *gene (AATGCTTACCGTGTGACCC; CATGCAGATTCAAAGACCAGC), murine *p53 *gene (TGGAGAGTATTTCACCCTCAAGA; CTCCTCTGTAGCATGGGCATC), m*Cdkn1a *(GATCCACAGCGATATCCAGAC; ACCGAAGAGACAACGGCACAC), m*Mdm2 *(TGTCTGTGTCTACCGAGGGTG; TCCAACGGACTTTAACAACTTCA). Real-Time quantitations were performed using the ABS *7500 Fast *Real-Time PCR System (Applied Biosystems). Fluorescence threshold value was calculated using the 7000 system SDS-software. The *Gapdh *gene (primers: GGTGAAGG TCGGTGTGAAC; GGGGTCTCGCTCCTGGAA) was used as a reference to compare the relative expression level of target genes.

### Immunofluorescence analysis

Primary neurons were grown on poly-L-Lysine (Sigma) coated coverslips. After washing in PBS, cells were fixed in 4% paraformaldehyde, washed and treated with 0,1 M glycine solution. Cells were permeabilized using 0,2% Triton X-100 and unspecific epitopes were blocked with 2% BSA solution. PrP^C ^was detected using POM1 antibody (1:200) and Alexa Fluor^® ^488-conjugated donkey anti-mouse secondary antibody (1:500; Invitrogen). For colocalization studies antibodies against PDI (rabbit; 1:100; Stressgen), GM130 (rabbit; 1:50; Abcam), and LAMP1 (rat; 1:50; Hybridoma Bank) were used to visualize ER, Golgi, and lysosomes, respectively. Secondary antibodies Alexa Fluor^® ^555 donkey anti-rabbit and anti-rat (Invitrogen) were diluted 1:500. Analysis was performed using an Improvision LiveCell Spinning Disk (Zeiss) or a TCS SP2 confocal microscope (Leica) and Volocity software (Perkin Elmer).

### Statistical analysis

Statistical comparison of Western blot quantifications and qPCR results between A10 cKO and littermate control samples was performed using Student's t-test with consideration of statistical significance at *p*-values < 0,05 (*) and <0,01 (**).

## Competing interests

The authors declare that they have no competing interests.

## Authors' contributions

The overall study was conceived and designed by HCA, BP, and MG, with important contributions from PS. HCA, JP, MAK, CB, DT, BP, and UB: performed the experiments. HCA, MAK, and CB: analysed the data. JP, PS, EJ, BP, UB, and BDS: contributed reagents, techniques or analysis tools. HCA and MG wrote the paper with substantial contribution from all co-authors. All authors read and approved the final manuscript.

## Supplementary Material

Additional file 1 **(A) Western blot analysis for premature (pADAM10) and mature ADAM10 (mADAM10; upper row) and β-actin (lower row) in neuronal cultures from *Prnp***^**0/0**^**, wt, *tg*a*20*, ADAM10 cKO and littermate controls at E14**. Levels of ADAM10 are dramatically reduced in ADAM10 cKO when compared to cultures from littermate controls or from mice overexpressing or lacking PrP^C^. (B) Western blot analysis for PrP^C ^after PNGase F treatment in neuronal lysates from *Prnp*^0/0^, wt, *tg*a*20*, ADAM10 cKO and littermate controls at E14 showing partially digested full-length prion protein and C1 fragment. (C) Shed PrP^C ^was immunoprecipitated (IP) from culture supernatants of primary neurons derived from *Prnp*^0/0^, wt, *tg*a*20*, ADAM10 cKO and littermate control mice (all from E14 embryos) and visualized by Western blot analysis for PrP^C^. Shed full-length PrP^C^, which shows a 2-3 kDa shift when compared to PrP^C ^in lysates, is only detectable in wt, *tg*a*20*, and littermate control neurons. Supernatants from ADAM10 cKO neuronal cultures contain virtually no shed full-length PrP^C^, whereas the soluble N1 fragment becomes detectable even in these ADAM10 cKO samples when POM2 is used for pull-down and detection. IgG light chain (IgG-LC) of capture antibody is detectable at 25 kDa. Click here for file
