# Supplementary figures and images for: Lack of a-disintegrin-and-metalloproteinase ADAM10 leads to intracellular accumulation and loss of shedding of the cellular prion protein in vivo
Source: Mol Neurodegener. 2011 May 27;6:36. doi: 10.1186/1750-1326-6-36 (PMC3224557; doi:10.1186/1750-1326-6-36)

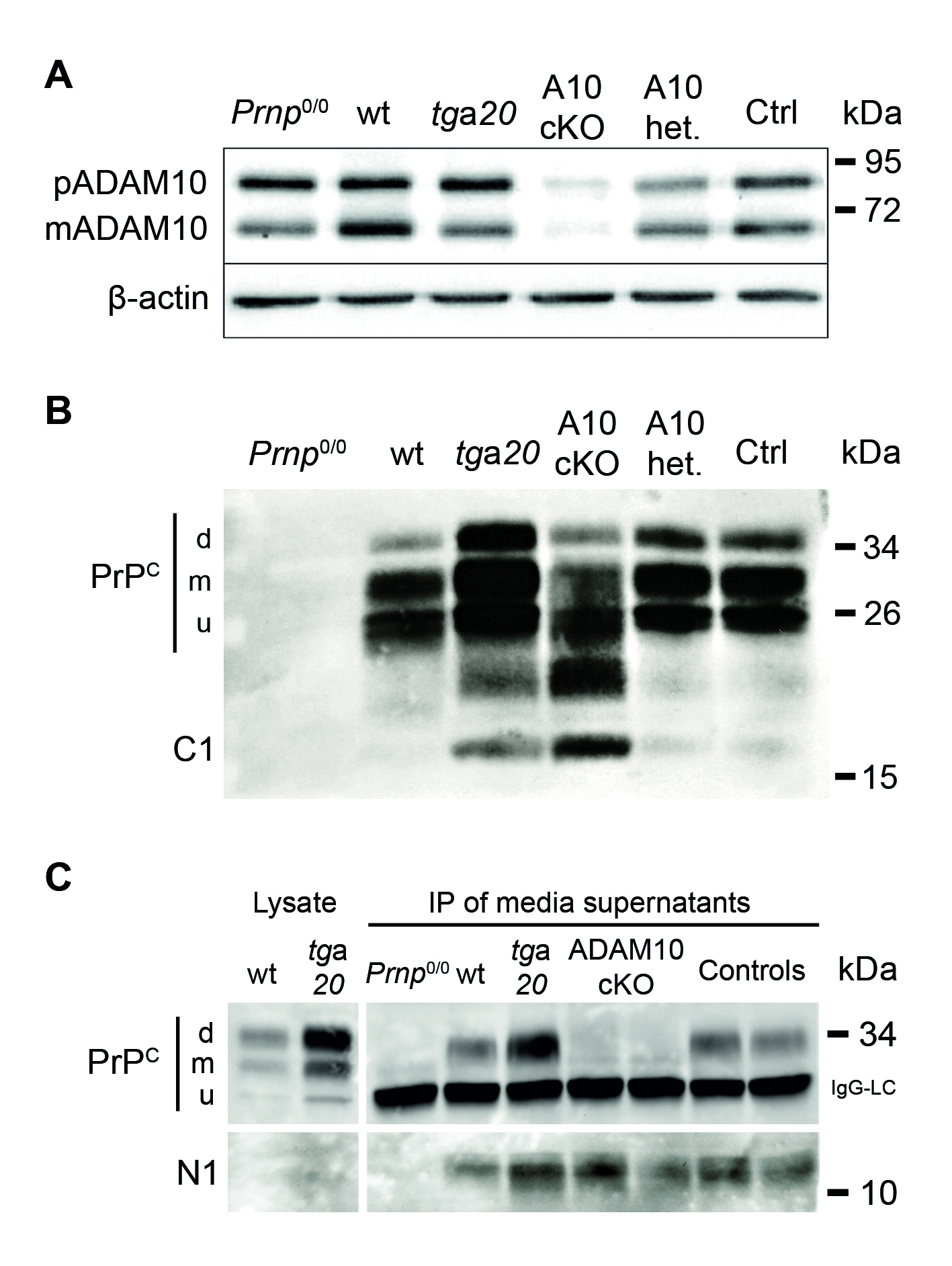

Supplement: Additional file 1 — (A) Western blot analysis for premature (pADAM10) and mature ADAM10 (mADAM10; upper row) and β-actin (lower row) in neuronal cultures from Prnp0/0, wt, tga20, ADAM10 cKO and littermate controls at E14. Levels of ADAM10 are dramatically reduced in ADAM10 cKO when compared to cultures from littermate controls or from mice overexpressing or lacking PrPC. (B) Western blot analysis for PrPC after PNGase F treatment in neuronal lysates from Prnp0/0, wt, tga20, ADAM10 cKO and littermate controls at E14 showing partially digested full-length prion protein and C1 fragment. (C) Shed PrPC was immunoprecipitated (IP) from culture supernatants of primary neurons derived from Prnp0/0, wt, tga20, ADAM10 cKO and littermate control mice (all from E14 embryos) and visualized by Western blot analysis for PrPC. Shed full-length PrPC, which shows a 2-3 kDa shift when compared to PrPC in lysates, is only detectable in wt, tga20, and littermate control neurons. Supernatants from ADAM10 cKO neuronal cultures contain virtually no shed full-length PrPC, whereas the soluble N1 fragment becomes detectable even in these ADAM10 cKO samples when POM2 is used for pull-down and detection. IgG light chain (IgG-LC) of capture antibody is detectable at 25 kDa. [file 1750-1326-6-36-S1.JPEG]
